# Supplementary material for: Real-time heart rate variability according to ambulatory glucose profile in patients with diabetes mellitus
Source: Front Cardiovasc Med. 2023 Nov 16;10:1249709. doi: 10.3389/fcvm.2023.1249709 (PMC10687410; doi:10.3389/fcvm.2023.1249709)
Supplement: Supplementary file 1 [file Table1.docx]

**Table 1. Baseline characteristics according to TIR in patients with DM**

| **Total patients = 38** |  |  |  |
| --- | --- | --- | --- |
| **Variable** | **TIR<70 % (n=13)** | **TIR>70 % (n=25)** | **P value** |
| Mean glucose level (mg/dL) | 241.1±60.3 | 129.3±27.3 | <0.001 |
| Age (years) | 64.3±6.2 | 66.2±6.1 | 0.161 |
| Sex (%), male | 6 (46.2) | 14 (56.0) | 0.734 |
| DM (%) | 13 (100) | 25 (100) | 1.000 |
| HTN (%) | 12 (92.3) | 18 (72.0) | 0.222 |
| Hyperlipidemia (%) | 11 (84.6) | 19 (76.0) | 0.689 |
| CAD (%) | 3 (23.0) | 9 (36.0) | 0.486 |
| CVA (%) | 2 (15.4) | 6 (24.0) | 0.689 |
| CHF (%) | 0 (0) | 2 (12.0) | 0.538 |
| CMP (%) | 1 (7.7) | 4 (16.7) | 0.638 |

*TIR indicates target in glucose range; DM, diabetes mellitus; HTN, hypertension; CAD, coronary artery disease; CVA, cerebrovascular accident; CHF, congestive heart failure; CMP, cardiomyopathy.

**Table 2. Baseline medicafions according to TIR in patients with DM**

| **Total patients = 38** | |  |  | |  | |  |
| --- | --- | --- | --- | --- | --- | --- | --- |
| **Variable** | | **TIR<70 % (n=13)** | **TIR>70 % (n=25)** | | **P value** | |  |
| **Medications** |  | | |  | |  | |
| BB (%) | 1 (7.7) | | | 9 (36.0) | | 0.118 | |
| CCB (%) | 9 (69.2) | | | 12 (48.0) | | 0.307 | |
| ARB/ACEi (%) | 7 (53.8) | | | 17 (68.0) | | 0.486 | |
| Diuretics (%) | 0 (0) | | | 7 (28.0) | | 0.072 | |
| Statin (%) | 11 (84.6) | | | 21 (84.0) | | 1.000 | |
| Aspirin/clopidogrel (%) | 7 (53.8) | | | 11 (44.0) | | 0.689 | |
| **DM medications** |  | | |  | |  | |
| Insulin (%) | 7 (53.8) | | | 7 (28.0) | | 0.163 | |
| Metformin (%) | 7 (53.8) | | | 16 (64.0) | | 0.728 | |
| Sulfonylurea (%) | 9 (69.2) | | | 12 (48.0) | | 0.307 | |
| sGLT inhibitor (%) | 5 (38.5) | | | 10 (40.0) | | 0.728 | |
| DPP-4 inhibitors (%) | 5 (38.5) | | | 11 (44.0) | | 1.000 | |

*TIR indicates target in glucose range; DM, diabetes mellitus; BB, beta-blocker; CCB, calcium channel blocker; ARB, angiotensin receptor blocker; ACEi, angiotensin converting enzyme inhibitor; sGLT inhibitor, sodium-glucose transport protein 2 inhibitor; DPP-4 inhibitor, Dipeptidyl peptidase 4 inhibitor.
